# The effects of oxygen in spinel oxide Li_1+x_Ti_2−x_O_4−δ_ thin films

**DOI:** 10.1038/s41598-018-22393-8

**Published:** 2018-03-05

**Authors:** Yanli Jia, Ge He, Wei Hu, Hua Yang, Zhenzhong Yang, Heshan Yu, Qinghua Zhang, Jinan Shi, Zefeng Lin, Jie Yuan, Beiyi Zhu, Lin Gu, Hong Li, Kui Jin

**Affiliations:** 1 Beijing National Laboratory for Condensed Matter Physics, Institute of Physics, Chinese Academy of Sciences, Beijing, 100190 China; 2 School of Physical Sciences, University of Chinese Academy of Sciences, Beijing, 100049 China; 3 Collaborative Innovation Center of Quantum Matter, Beijing, 100190 China

## Abstract

The evolution from superconducting LiTi_2_O_4-δ_ to insulating Li_4_Ti_5_O_12_ thin films has been studied by precisely tuning the oxygen pressure in the sample fabrication process. In superconducting LiTi_2_O_4-δ_ films, with the increase of oxygen pressure, the oxygen vacancies are filled gradually and the *c-*axis lattice constant decreases. When the oxygen pressure increases to a certain critical value, the *c*-axis lattice constant becomes stable, which implies that the sample has been completely converted to Li_4_Ti_5_O_12_ phase. The two processes can be manifested by the angular bright-field images of the scanning transmission electron microscopy techniques. The transition temperature (*T*_*ch*_) of magnetoresistance from the positive to the negative shows a nonmonotonic behavior, *i*.*e*. first decrease and then increase, with the increase of oxygen pressure. We suggest that the decrease *T*_ch_ can be attributed to the suppressing of orbital-related state, and the inhomogeneous phase separated regions contribute positive MR and thereby lead to the reverse relation between *T*_ch_ and oxygen pressure.

## Introduction

In the research on oxide superconductors, the oxygen always plays an important role in the superconductivity and their normal state behaviors^1,2^. In copper oxide high- critical temperature (*T*_c_) superconductors, such as Nd_2-x_Ce_x_CuO_4±δ_^3–5^, Pr_2−*x*_Ce_*x*_CuO_4±δ_^6–8^ and YBa_2_Cu_3_O_7−δ_^9–11^, *T*_*c*_ can be greatly improved in a large range by adjusting the oxygen content during the annealing process, as well as the titanium oxide systems, such as SrTiO_3_^12,13^ and TiO^14,15^. Oxygen has a strong effect not only on superconductivity, but also on many other properties. For instance, the antiferromagnetism^16^ and the charge density wave^17^ can also be tuned by the oxygen vacancies. Furthermore, the doping and disorder effects induced by oxygen vacancies can cause obviously change on Hall resistance and magnetoresistance (MR) behaviors in the normal state^5,6,18^. Studying the oxygen effects is of great help to understand the mechanism of superconductivity, transport and other properties of the oxide superconductor^9,19,20^.

Among hundreds of spinel oxides, the metallic lithium titanate LiTi_2_O_4_ is the only known oxide superconductor, which *T*_c_ is as high as 13.7 K^21^, discovered by Johnston *et al*. in 1973^22^. Previous studies have disclosed that LiTi_2_O_4_ is a BCS *s*-wave superconductor with intermediate electron-phonon coupling (λ_el-ph_ ~ 0.65)^23,24^. Nevertheless, an enhanced density of states has been unveiled by magnetic susceptibility^25^ and specific heat measurements^23^, indicating that *d-d* electronic correlations cannot be ignored in this system. Meanwhile, due to the mixed-valence of Ti ions in the frustrated Ti sublattice, LiTi_2_O_4_ exhibits complicated spin-orbit fluctuations, which is evidenced by the resonant inelastic soft-x-ray scattering^26^, nuclear magnetic resonance^27^ and magnetic susceptibility measurements^25^. Very recently, electrical transport and tunneling spectra measurements on high quality epitaxial [001]-oriented LiTi_2_O_4_ films have revealed an orbital-related state below ~50 K, confirmed by a twofold in-plane angular dependent MR, positive MR as well as the relation Δ ~−B^2^ ^28^.

Interestingly, by tuning the oxygen in the process of sample deposition, the phase of the thin film changes from LiTi_2_O_4-δ_ to Li_4_Ti_5_O_12_ along with the superconductor-insulator phase transition^29^. However, this transition seems to happen abruptly, which hinders us from understanding the nature of the transition. Previous work on LiTi_2_O_4_ polycrystals has disclosed that the existence of oxygen-site distortion induces prominent changes in the electronic states near *E*_F_^30^_._ In addition, tunneling spectra experiments on LiTi_2_O_4_ films of different orientations reveal an anisotropic electron-phonon coupling in this system, which is regarded to originate from the *Jahn-Teller* distortions enhanced by oxygen vacancies^31^. Nevertheless, it is still unclear what happens in the microstructure of the sample during the transition from LiTi_2_O_4_ to Li_4_Ti_5_O_12_. Moreover, the mechanism of the oxygen effects on superconductivity of LiTi_2_O_4_ has never been investigated, as well as the transport behaviors in the normal state. Therefore, it is worthy of tuning the oxygen pressure (*P*_O2_) in the process of film deposition to clarify these questions.

In this work, we carefully manipulated the transition from LiTi_2_O_4-δ_ to Li_4_Ti_5_O_12_ thin films by adjusting the *P*_O2_ in the process of pulsed laser deposition (PLD). First, the high quality LiTi_2_O_4-δ_ superconducting thin films can be obtained in the high vacuum environment. Tuning the *P*_O2_ from 10^−7^ to 10^−4^ Torr, the *c-*axis lattice constant gradually decreases, indicating that the filling of oxygen vacancies dominates in this process. Second, when *P*_O2_ is higher than 10^−4^ Torr, the *c*-axis lattice constant stops to decrease, indicating the finish of transition from LiTi_2_O_4-δ_ to Li_4_Ti_5_O_12_ phase. These two processes can be revealed from the angular bright-field images (ABF) of LiTi_2_O_4-δ_ and Li_4_Ti_5_O_12_ by the scanning transmission electron microscopy (STEM) techniques. In addition, the temperature (*T*_*ch*_) of MR from the positive to the negative shows a nonmonotonic behavior, *i*.*e*. first decrease and then increase, with the increase of *P*_O2_. Combined with the electron energy-loss spectroscopy (EELS) measurements, we suggest that the decrease of *T*_*ch*_ under lower *P*_O2_ stems from the suppression of orbital-related state via filling the oxygen vacancies, and the increase of *T*_*ch*_ under higher *P*_O2_ is due to the phase separation in some regions, which dominates the positive MR (p-MR).

## Results and Discussion

**Figure 1:**
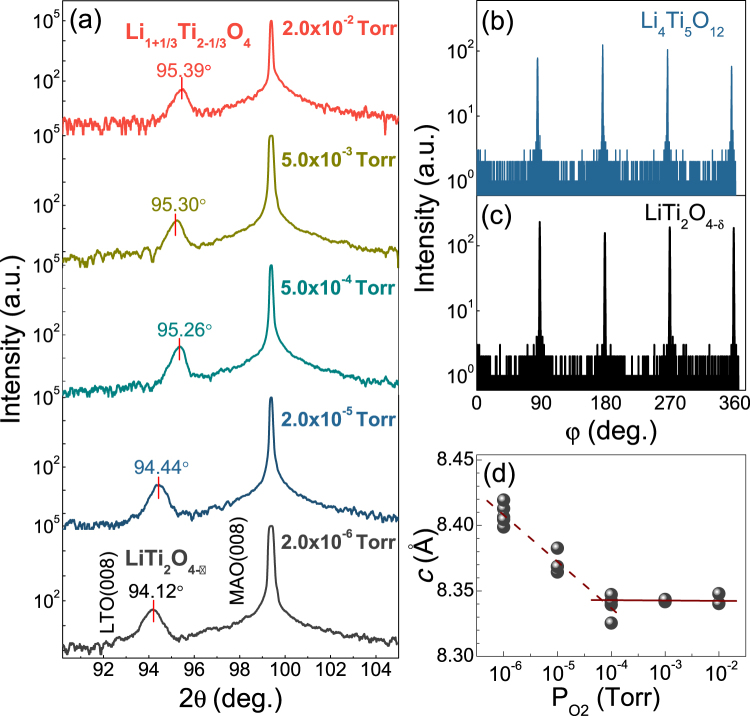
(**a**) The *θ–2θ* XRD spectra of epitaxial Li_1+x_Ti_2-x_O_4-δ_ (0 ≤ × ≤ 1/3) thin films grown on (001) MAO substrates at different *P*_O2_. (**b**) The *φ*-scan measurements of LiTi_2_O_4-δ_ and Li_4_Ti_5_O_12_ thin films on MAO (001) in the (404) reflection. (**c**) The lattice constant along *c* axis of the samples versus *P*_O2_.

The *θ*–2*θ* XRD spectra of (001) Li_1+x_Ti_2-x_O_4-δ_ (0 ≤ × ≤ 1/3) samples grown in different *P*_O2_ are shown in Fig. 1(a). The (001)-oriented LiTi_2_O_4-δ_ thin films are achieved when the films are deposited under *P*_O2_ ≤ 10^−6^ Torr. Instead, the (001)-oriented Li_4_Ti_5_O_12_ thin films are formed at *P*_O2_ > 10^−4^ Torr. The XRD patterns of the samples in different *P*_O2_ are quite similar except that the diffraction peaks gradually shift to higher angle in the LiTi_2_O_4-δ_ films at larger *P*_O2_. In order to check the crystallization quality of the thin films, we also perform *φ*-scan. In Fig. 1(b), the *φ*-scans of (404) plane of both LiTi_2_O_4-δ_ and Li_4_Ti_5_O_12_ samples display four-fold symmetry with uniformly distributed peaks. From the *θ*–2*θ* XRD spectra, we can extract the value of the out-of-plane lattice constant (*c-axis*) as a function of the *P*_O2_. As seen in Fig. 1(c), when *P*_O2_ < 10^−4^ Torr, *c* gradually decreases with increasing *P*_O2_. However, when *P*_O2_ is higher than 10^−4^ Torr, *c* is saturated, indicating the complete formation of Li_4_Ti_5_O_12_ phase. As a result, a phase transition from LiTi_2_O_4-δ_ to Li_4_Ti_5_O_12_ has been successfully achieved by tuning *P*_O2_ during the sample deposition.

**Figure 2:**
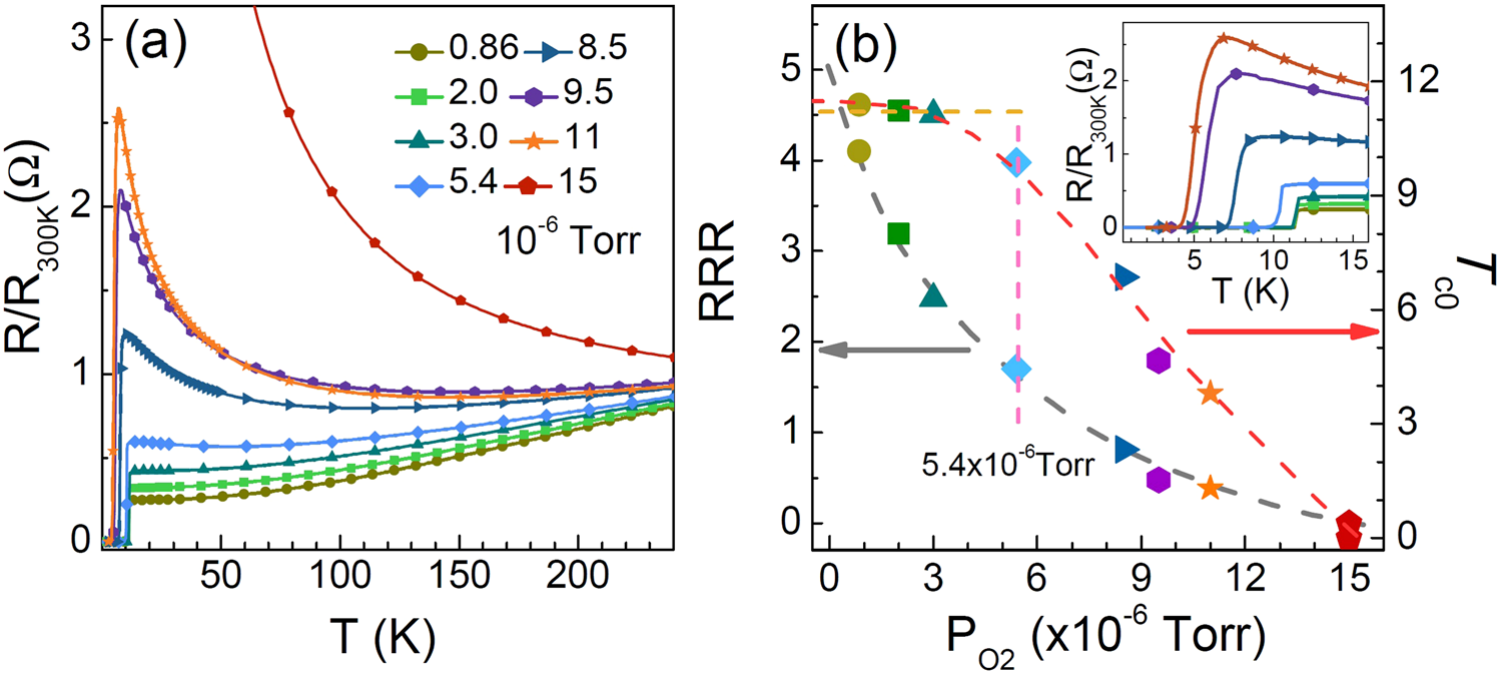
(**a**) The *R*-*T* curves of Li_1+x_Ti_2-x_O_4-δ_ (0 ≤ × ≤ 1/3) thin films grown on (001) MAO substrate with different *P*_O2_ during the deposition. (**b**) The *P*_O2_ dependence of *RRR* and *T*_c0_ of the films in (**a**) are plotted. The gray and red dashed lines are used to guide eyes. *T*_c0_ is defined as the temperature where resistance is lower than 10^−6^ Ohm. Inset: zoom the *R*-*T* curves in Fig. 2(a) at low temperature range.

In order to further study the effects of oxygen on superconducting state and normal state, we systematically measured the resistances of various thin films from LiTi_2_O_4-δ_ to Li_4_Ti_5_O_12_. The R-T curves of the LiTi_2_O_4-δ_ thin films with different oxygen pressures are shown in Fig. 2(a). Increasing the *P*_O2_ during the deposition, the samples undergo a transition from metal to insulator in the normal state. In Fig. 2(b), the residual resistivity ratio (*RRR*) decreases monotonically with increasing *P*_O2_. Here, the *RRR* is defined as the ratio of room temperature resistivity to the resistivity of *T*_*c*_°^nset^, *i*.*e*. *R* (300 K)/*R* (*T*_*c*_°^nset^), where the *T*_*c*_^onset^ is the critical temperature at the beginning of superconducting transition. We plot the dependence of *T*_c0_ on *P*_O2_ as seen in Fig. 2(b), and the *T*_*c0*_ of the LiTi_2_O_4-δ_ thin films is quite stable at *P*_O2_ < 5.4 × 10^−6^ Torr, whereas it drops rapidly when *P*_O2_ > 5.4 × 10^−6^ Torr.

**Figure 3:**
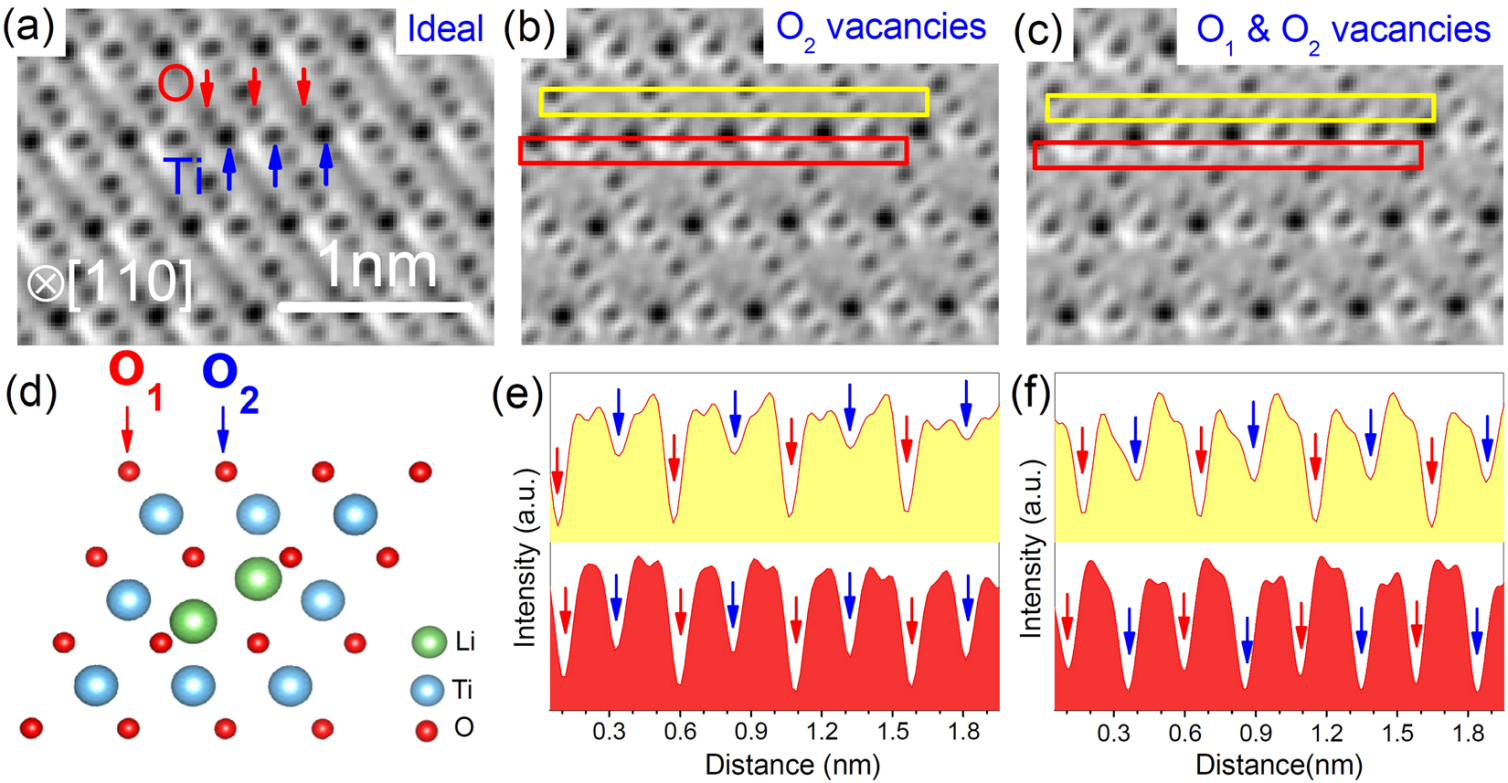
ABF images of LiTi_2_O_4-δ_ thin film in (**a**) pristine, (**b**) O_2_ vacancy and (**c**) O_1_ & O_2_ vacancy regions. (**d**) Structure model of LiTi_2_O_4_ projected along [110] direction, where the atomic positions of both O_1_ and O_2_ oxygen are labeled by red and blue arrow, respectively. (**e**) and (**f**) Line profiles of ABF contrast with filled yellow and red color, obtained from the corresponding yellow and red areas in (**b**) and (**c**), respectively. Atomic positions of O_1_ and O_2_ are also labeled by red and blue arrows, respectively. Note that the lower panel in (**e**) exhibits contrast between O_1_ (red arrows) and O_2_ (blue arrows) close to the ideal structure.

To find out the microstructure evolution from LiTi_2_O_4-δ_ to Li_4_Ti_5_O_12_, we have carried out atomic-resolution STEM measurements on these high-quality samples. Figure 3 shows the ABF images along the [110] direction and the corresponding line profiles in different regions with different types of oxygen vacancies. In Fig. 3(a), the O columns, as indicated by red arrows, are imaged as dark spots due to the absorption nature of the ABF contrast, and the contrast of the Ti columns as indicated by the blue arrows is darker than the O columns based on the ~Z^1/3^ contrast mechanism where Z is the atomic number. Thus, in the pristine regions the contrast of O_1_ and O_2_ is of approximate equal darkness, and oxygen vacancies as shown in Fig. 3(b) and (c), are imaged as light gray spots. Here, we divided the positions of oxygen atoms into two types, *i*.*e*. O_1_ and O_2_ (see Fig. 3(d)) to describe clearly the distribution of oxygen vacancies, as shown by the red and blue arrows. To visualize the oxygen vacancies clearly, we extracted the line profiles on the oxygen rows as indicated by the yellow and red rectangles.

From the line profile of ABF contrast in Fig. 3(e), we can find that the depth of the ABF contrast valley (darkness) at O_2_ positions is lower than that at O_1_ as seen in the [110] direction, which means some vacancies exist in the O_2_ sites. Similarly, some vacancies at O_1_ and O_2_ exist in another region as shown in Fig. 3(f). However, these oxygen vacancies have not been observed in the Li_4_Ti_5_O_12_ samples^32^. It is known that the LiTi_2_O_4-δ_ exhibits serious aging effects in forms of polycrystal and single crystal^30^. The LiTi_2_O_4-δ_ thin films, especially the one deposited in the higher vacuum, are much more stable. It is reasonable to speculate that the samples in higher vacuum will contain more oxygen vacancies. Increasing oxygen pressure will fill these oxygen vacancies and finally turn the superconducting phase to insulating Li_4_Ti_5_O_12_.

The phase evolution with *P*_O2_ should inevitably make difference in the electronic states. In LiTi_2_O_4-δ_, one concern is about the orbital-related state. Normally, the formation of the orbital order results from the band split near the Fermi level. As for LiTi_2_O_4-δ_, the distortion of Ti-O octahedron leads the splitting of Ti 3d band to e_g_ and t_2g_ band^33^, and the orbital-related state is expected to exist. Although it has been unveiled in previous work, it remains unclear in the origin^25^. One of the evidence is the crossover from the negative MR (n-MR) to the p-MR at *T*_*ch*_ ~ 50 K in the normal state. Entering the superconducting state, the orbital-related state interacts with Cooper pairs and results in an unexpected relation between the superconducting gap and the applied magnetic field, *i*.*e*. Δ~ −B^2^. This relation implies the coexistence of the superconducting state and the orbital-related state. Therefore, it is deserved to clarify how the oxygen makes the influence on these two states.

**Figure 4:**
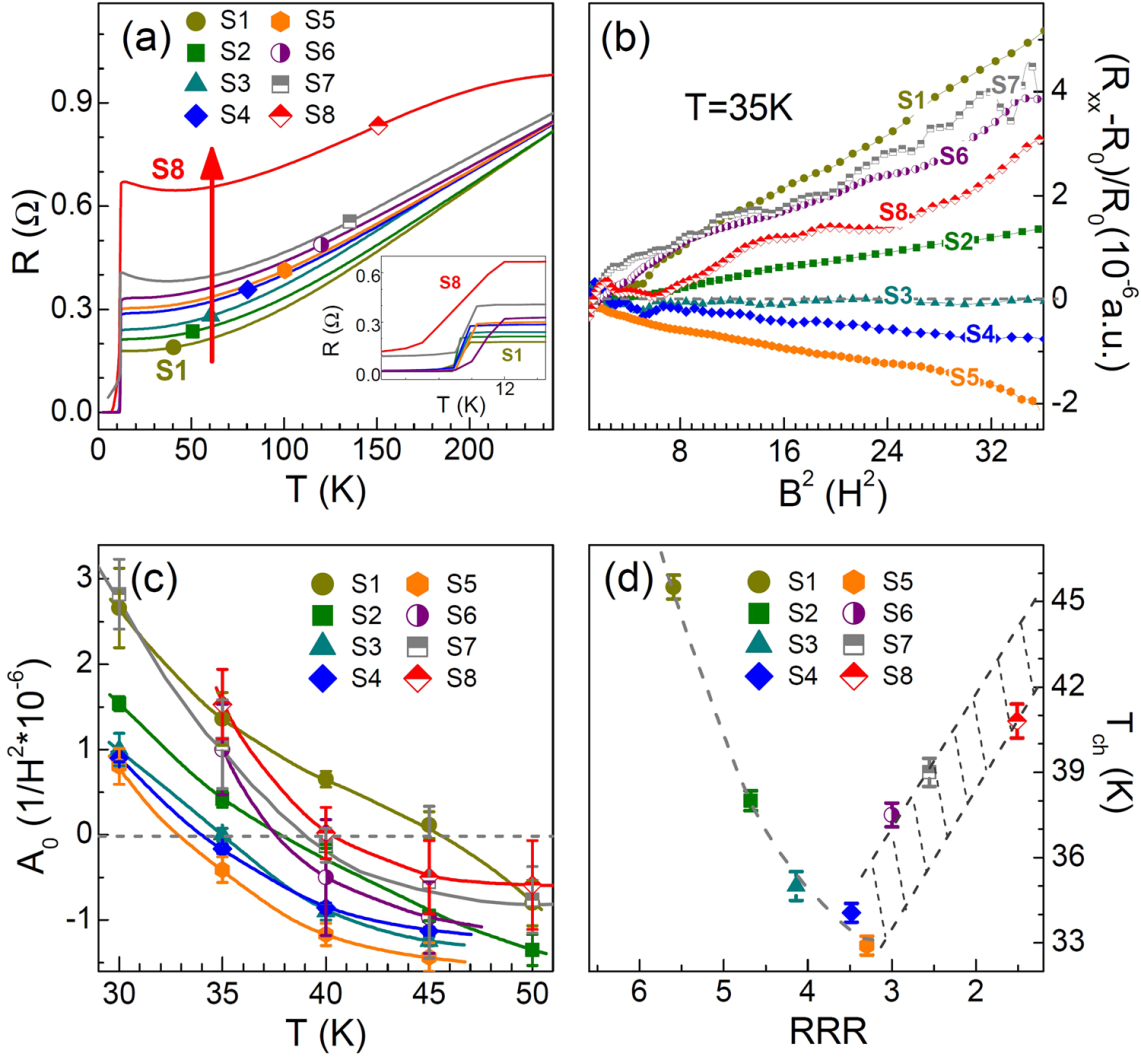
(**a**) The *R*-T curves with increase of *P*_O2_ are defined as S1–S8 in sequence. The inset is the zoom of *R*-T curves. (**b**) The field dependence of MR of S1–S8 grown on (001) MAO substrates at 35 K. (**c**) The slope value *A*_0_ of MR can be obtained for various samples at different temperatures. With temperature increasing, the value of *A*_0_ changes from the positive to the negative. (**d**) The relationship between *RRR* and *T*_ch_. The gray dashed line is used to guide eyes. The shadow areas represent the uncertainties in defining *T*_ch_ due to the impurities.

To clarify this issue, we finely tune the *P*_O2_ around 10^−6^ Torr to avoid the Li_4_Ti_5_O_12_ phase. Then, we focus on the effects of *P*_O2_ on R and MR. In the precise tuning process, the vacuum value is not a good scale due to the limitation of the vacuum gauge. Fortunately, the *RRR* decreases monotonically with the increase of oxygen pressure, which can reflect the trend of *P*_O2_ and the oxygen defects as discussed above. Thus, we use *RRR* to index the samples, named S1 to S8 with *RRR* in the range between 5.6 and 1.5. As shown in Fig. 4(a), the *T*_c_ seems unchanged in the tuning range. For samples S1 to S5, the MR at 35 K changes from positive to negative as seen in Fig. 4(b). By fitting the MR with the Kohler’s formula, *i*.*e*. MR ~*A*_0_B^2^ ^2^, the slope *A*_0_ can be obtained for these samples at different temperatures. In Drude model, *A*_0_ is proportional to *μ*^2^ (*i*.*e*. *μ* = eτ/*m*) with *μ* the mobility, τ the relaxation time and *m* the electron mass. With the increase of temperature, the value of *A*_0_ decreases from positive to negative as seen in Fig. 4(c). A negative mobility cannot be understood in this simplified model, and the n-MR is interpreted as the suppression of spin-orbital fluctuations in this system^28^. The *T*_ch_ from p-MR to n-MR is extracted from Fig. 4(c) and plotted in Fig. 4(d). From S5 to S1, *T*_ch_ gradually increases with the increase of *RRR*.

However, further reducing the *RRR*, the relation between *T*_ch_ and *RRR* will be broken. For instance, the MR becomes stronger for the samples deposited under higher *P*_O2_, *e*.*g*. S6–S8, and thus the *T*_ch_ goes up. In this regime, the formation of Li_4_Ti_5_O_12_ phase may lead to more boundaries in phase separated samples. Such inhomogeneity in the magnetic field usually exhibits strong p-MR^34,35^. For the samples S1–S5, the p-MR below *T*_*ch*_ mainly origins from the orbital-related state since the LiTi_2_O_4-δ_ phase dominates the transport^25^. We speculate that filling the oxygen vacancies seems to suppress the p-MR but in fact the orbital-related state.

**Figure 5:**
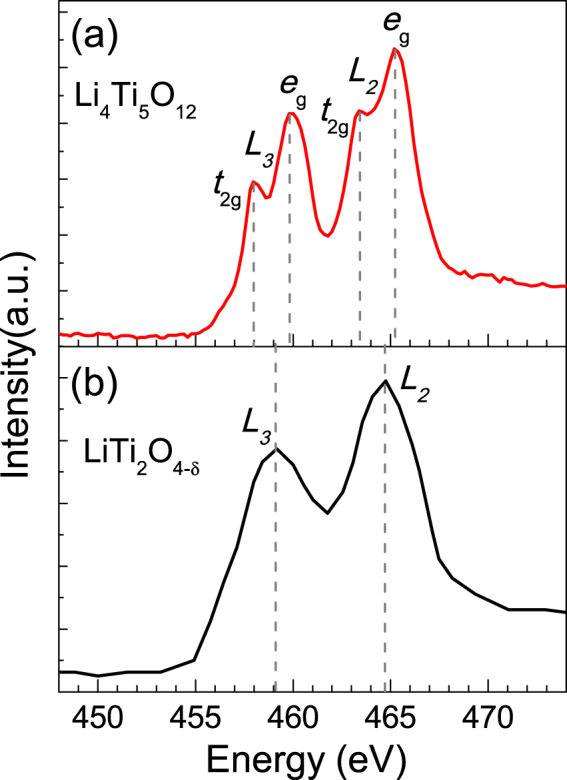
(**a**) The EELS profiles for Ti *L*_2,3_ edges of the Li_4_Ti_5_O_12_ thin film. The *L*_2_ and *L*_3_ edges split into four peaks. (**b**) The EELS profiles for Ti *L*_2,3_ edges of the LiTi_2_O_4-δ_ thin film. Only two peaks appear of *L*_2,3_ edges.

In order to verify this assumption, we should evaluate the effects of oxygen vacancies on Ti valance. Although oxygen vacancies have been detected by STEM, the content of oxygen vacancies cannot be quantified. Therefore, we collected EELS profiles of both LiTi_2_O_4-δ_ and Li_4_Ti_5_O_12_ films. As seen in Fig. 5, the Ti *L*_2,3_ edges, from 2*p*_1/2_ and 2*p*_3/2_ to 3*d* orbits respectively, split into two peaks in Li_4_Ti_5_O_12_, but not in LiTi_2_O_4-δ_. Usually, the splitting of *L*_2,3_ is attributed to the degeneracy lifting of Ti 3*d* orbits by the crystal field.

The missing split of peaks in LiTi_2_O_4-δ_ EELS may origin from two reasons. First, the energy gap between *e*_g_ and *t*_2g_, named Δ_e–t_, is too small to be discernable in EELS. According to the band calculations, Δ_e-t_ equals to 2.1 eV and 2.4 eV for the ideal structure LiTi_2_O_4_ and Li_4_Ti_5_O_12_, respectively^36^. Moreover, four peaks were also observed in Li_7_Ti_5_O_12_, where Δ_e-t_ equals to 1.8 eV^32^. Considering the existence of oxygen vacancies, which may further distort the Ti-O octahedrons, we do not expect a smaller crystal field. Therefore, the change of Δ_e–t_ cannot account for the discernable peak splitting in LiTi_2_O_4-δ_. Second, the valance of Ti in ideal LiTi_2_O_4_ is +3.5. If large numbers of oxygen vacancies exist in LiTi_2_O_4-δ_, the Ti^3.5+^ will transform to Ti^3+^. In this condition, the electrons on *t*_2g_ band increase, and thereby the hoping possibility from Ti 2*p* to *t*_2g_ is reduced due to the Pauli Exclusion Principle. Consequently, oxygen vacancies will smear out the peaks of Ti 2*p* to *t*_2g_ in EELS.

Based on the EELS results, we can give a reasonable explanation for the suppression of the orbital-related state by filling oxygen vacancies. In general, the formation of the orbital order results from the band split near the Fermi level. As for LiTi_2_O_4_, crystal field splits Ti 3*d* bands to e_g_ and t_2g_ bands^33^. The oxygen vacancies in LiTi_2_O_4-δ_ system, on the one hand, enhance the distortion of Ti-O octahedrons, on the other hand, dope electrons to enhance the electron correlations, which are beneficial to the formation of the orbital-related state. With the filling of oxygen vacancies, the valence of Ti increases and some of Ti sites become empty states, thereby weaken the orbital-related state.

Compared to the obviously suppressed orbital-related state, the *T*_*c0*_ of the LiTi_2_O_4-δ_ thin films is quite stable at *P*_O2_ < 5.4 × 10^−6^ Torr. Actually, the O 2*p* bands are far below the Fermi level with weak *p*-*d* hybridizations^30,33^. Although the oxygen vacancies induce doping effect and influence on the splitting of Ti 3*d* bands by the crystal field, the density of states near Fermi surface may not change obviously, and thus the *T*_*c0*_ remains the same.

In conclusion, we studied the evolution from LiTi_2_O_4-δ_ to Li_4_Ti_5_O_12_ with increasing oxygen pressure during the thin film deposition. By transport and STEM measurements, we have disclosed that there are two processes happened during the evolution, *i*.*e*. the filling of oxygen vacancies and the forming of Li_4_Ti_5_O_12_. The EELS results of the LiTi_2_O_4-δ_ and Li_4_Ti_5_O_12_ samples provide the evidence that the orbital-related state is suppressed by the filling of oxygen vacancies. The evolution of electronic states by adjusting the oxygen content gives an insight into the interaction between the orbital-related state and the superconductivity in LiTi_2_O_4-δ_.

## Methods

The (00*l*)-oriented Li_1+x_Ti_2-x_O_4-δ_ (0 ≤ × ≤ 1/3) thin films are grown on (00 *l*) MgAl_2_O_4_ (MAO) substrates by PLD with a *K*_r_F excimer laser (λ = 248 nm). Before the deposition, the MAO substrates are annealed at 1000 °C for 5 hours in the air^37,38^ to obtain the smooth surface. The sintered Li_4_Ti_5_O_12_ ceramic target is used to fabricate the films, with pulse frequency of 4 Hz, energy density of 1.5 J/cm^2^, and deposition temperature of ~700 °C. The deposition rate is determined by measuring the thickness of ultra-thin films using X-ray reflectivity analysis. In this study, we fix the film thickness ~150 nm. After the deposition, all the thin films are quenched to the room temperature *in situ*.

X-ray diffraction (XRD) is employed to characterize the phase and crystalline quality of Li_1+x_Ti_2-x_O_4-δ_ (0 ≤ × ≤ 1/3) thin films. The microstructure is detected by the spherical aberration-corrected scanning transmission electron microscopy techniques (Cs-STEM). The transport properties are measured by the Quantum Design Physical Property Measurement System (PPMS) with the temperature down to 2 K and magnetic field up to 9 T. Samples are etched into Hall bar by the UV lithography and Ar plasma etching technology for the measurement of the resistance properties.
